# Enhancing randomized clinical trials with digital twins

**DOI:** 10.1038/s41540-025-00592-0

**Published:** 2025-10-03

**Authors:** Hossein Akbarialiabad, Amirmohammad Pasdar, Dédée F. Murrell, Mehrnaz Mostafavi, Farhan Shakil, Ehsan Safaee, Sancy A. Leachman, Alireza Haghighi, Michelle Tarbox, Christopher G. Bunick, Ayman Grada

**Affiliations:** 1 University of Utah, Salt Lake City, UT USA; 2 Department of Dermatology, St George Hospital, Sydney, NSW Australia; 3 Faculty of Medicine, UNSW Medicine, University of New South Wales, Sydney, NSW Australia; 4 Missouri Baptist Medical Center, St. Louis, USA; 5 Washington University in St. Louis School of Medicine, St. Louis, USA; 6 School of Computing and Information Systems, University of Melbourne, Melbourne, VIC Australia; 7 Faculty of Allied Medicine, Shahid Beheshti University of Medical Sciences, Tehran, Iran; 8 George Herbert Walker School of Business & Technology, Webster University, Saint Louis, MO USA; 9 Faculty of Medicine, Shahed University, Tehran, Iran; 10 Division of Genetics, Department of Medicine, Brigham and Women’s Hospital, Harvard Medical School, Boston, MA USA; 11 Department of Medicine, Brigham and Women’s Hospital, Harvard Medical School, Boston, MA 02115 USA; 12 The Broad Institute, 415 Main St, Cambridge, MA 02142 USA; 13 Department of Dermatology, Texas Tech University Health Sciences Center, Lubbock, TX USA; 14 Department of Dermatology and Program in Translational Biomedicine, Yale School of Medicine, New Haven, CT USA; 15 Department of Dermatology, Case Western Reserve University School of Medicine, Cleveland, OH USA

**Keywords:** Diseases, Business and industry

## Abstract

Digital twins (DTs) can transform randomized clinical trials by improving ethical standards, including safety, informed consent, equity, and data privacy. They also enhance trial efficiency by enabling early detection of adverse events and streamlined design. This paper explores the role of DTs in personalized medicine, from pre-clinical research to post-marketing, while addressing technological, legal, and ethical challenges in implementation.

## Introduction

In medicine and other specialties, healthcare disparities may still prevail partially due to the determinants of health (DoH). As stated by the World Health Organization (WHO), the determinants of health consist of factors that affect people, giving rise to their health outcomes, including the social and economic environment, the physical environment, and individual characteristics and behaviors^1,2^. Electronic health records (EHRs) typically lack proper documentation of DoH data, which can restrict the depth of research^3,4^.

Clinical research and trials can inform public health initiatives. This includes epidemiological studies investigating patterns, causes, and effects of health and disease in specific populations. Observational studies, such as cohort studies, can provide insights into the burden of disease and real-world evidence on the effectiveness of therapies. Consequently, scientists must stay current with clinical research methodologies and trial protocols^5^. Interventions in illness management, such as therapy, prevention, and diagnosis, are the focus of clinical trials. The most reliable and trustworthy data comes from randomized controlled trials (RCTs), regarded as the gold standard because of their capacity to reduce bias. Research trials with a control group do have certain disadvantages.

Because RCTs operate under tightly controlled conditions, their findings often do not reflect everyday clinical practice. Restrictive eligibility criteria frequently exclude essential subgroups, and enrolling vulnerable patients, such as children, pregnant women, and individuals with mental impairments, presents additional ethical and regulatory challenges that further limit participant diversity^6^. Two main factors, therefore, limit external validity: (a) systematic under-representation of diverse demographic and clinical groups and (b) variation in treatment effects across subgroups^7^. Together, these factors make it challenging to generalize trial outcomes to the broader patient population and highlight the need for complementary approaches that can better forecast real-world effectiveness.

Generalizability is usually confirmed only after approval, through Phase-4 trials designed for specific subgroups, such as the ORAL Surveillance study that assessed the safety of tofacitinib in older rheumatoid-arthritis patients^8,9^. Real-world observational work offers a complementary perspective, like a Medicare claims analysis that compared bleeding risk with dabigatran versus warfarin^10^. Recruiting underrepresented patients, who make these studies truly informative, remains challenging as a recent systematic review of 43 reports identified ongoing logistical, cultural, and trust barriers, noting that minority groups comprised less than 2% of participants in many neuro-oncology trials^11^.

Beyond scientific impact, these delays also incur substantial economic costs: industry analyses estimate that each month of slowed enrollment can add roughly USD 500,000 in extra trial expenses and unrealized revenue^12^.

Digital twins (DTs) offer a promising approach by providing dynamic, individualized models that replicate specific patient populations that continuously update with real-time data. Many of these in silico representations leverage deep generative models to create synthetic patient cohorts that replicate the underlying structure of real-world populations. Integrating these synthetic cohorts can enhance the development and validation of DTs, improving their applicability in clinical settings. While DT synthesis aims to integrate diverse patient characteristics, its accuracy depends on the quality and representativeness of the data used to generate these models. Since DTs are often constructed using trial participant data, they may still inherit the same generalizability limitations as traditional RCTs. However, rigorous validation techniques and data augmentation strategies can help mitigate these constraints, improving the applicability of DT-driven research to broader patient populations. Notably, digital twin synthesis can integrate the multifaceted characteristics of various patient populations by constructing cohorts that accurately reflect the distribution of relevant covariates^13^.

DTs and AI virtual-assistant technologies are already progressing from concept to clinical application. A multicenter RCT launched in 2022, the inEurHeart trial, enrolled 112 patients to compare AI-guided ventricular tachycardia ablation planned on a cardiac DT with standard catheter techniques^14^. Early clinical evaluations of this platform report 60% shorter procedure times and a 15% absolute increase in acute success rates. Similarly, a 12-week RCT involving 112 older adults with type 2 diabetes showed that a smart-speaker-based virtual assistant lowered HbA1c by 0.48%, reduced mental distress scores, and improved self-care adherence compared to usual care^15^. These initial data demonstrate that DT- and AI-augmented interventions can produce measurable clinical benefits and suggest future RCT designs that achieve strong evidence with smaller, more diverse cohorts^16^. In addition, AI algorithms can analyze extensive datasets, such as electronic health records and patient registries, to identify individuals who meet specific eligibility criteria for clinical trials. Digital twins provide a patient-specific simulation platform for medical research that mimics disease activity and adverse reactions to investigational treatments^17^. With a virtual environment reproducing real-life situations, digital twins enable researchers to run experiments, test hypotheses, and create optimized drug candidates in drug discovery contexts^18^. Furthermore, concerning precision health, digital twins can help customize therapeutic intervention individually based on the patient’s characteristics, phenotype, endotype, and socio-economic constraints^19,20^. For instance, in oncology, digital twins are used to understand tumor dynamics and personalize cancer care, integrating medical imaging with mathematical modeling to tailor interventions^21,22^. This commentary on the role of digital twins in healthcare begins with presenting the challenge of incorporating DoHs into RCTs, more notably in precision health situations.

## Framework of AI-generated digital twins in RCTs

**Fig. 1:**
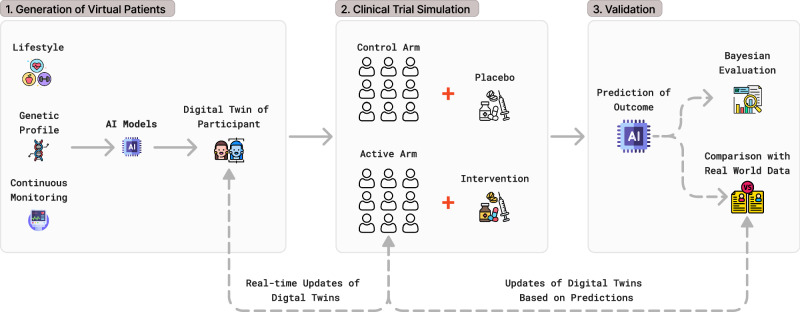
AI-driven digital twins framework in clinical trials. 1. Virtual patient generation: AI models use lifestyle, genetic, and monitoring data to create and update digital twins. 2. Trial simulation: DTs are assigned to control (placebo) and active (intervention) arms to simulate trial outcomes. 3. Validation: Predictions are evaluated using Bayesian analysis and compared with real-world data to refine the models. This figure was created using the free Figma application. All icons were sourced from Flaticon.com and are used under a free license with attribution.

To understand how digital twin technology enhances clinical trials, Fig. 1 presents a schematic framework of an AI-driven DTs work^23^.

1. Data collection and generation of virtual patients: The creation of AI-generated digital twins in clinical trials begins with the collection of comprehensive patient data. Baseline clinical information, including symptoms, biomarkers, imaging data, genetic profiles, and lifestyle factors, is gathered from trial participants. This data is then augmented with historical control datasets derived from previous clinical trials, disease registries, and real-world evidence studies. By integrating these diverse sources, AI models can generate synthetic patient profiles that accurately capture the variability of real-world populations. These virtual patients serve as the foundation for subsequent trial simulations and predictive analyses.
2. Simulation of virtual cohorts: Once virtual patients are created, AI models can be used in two complementary ways: (a) as synthetic controls that replace or reduce the number of real-world placebo groups, and (b) as virtual recipients of experimental therapies. For the synthetic-control approach, each real participant is paired with a digital twin whose progression is projected under standard care, offering comparator data without exposing additional patients to a placebo. The resulting virtual control group thus mirrors the natural course of the disease with routine management. Conversely, the virtual treatment group is generated by adding the expected biological effects of the investigational drug, inferred from preclinical and earlier clinical data. Employing digital twins for both groups enables researchers to explore efficacy and safety signals in silico, reduce sample size needs, shorten timelines, and prevent patients from unnecessary exposure to ineffective or potentially harmful treatments.
3. Predictive modeling and optimization of clinical trials: The AI-generated DTs undergo continuous refinement through predictive modeling techniques. AI-driven adaptive trial designs leverage virtual cohorts to optimize key trial parameters, such as dosing regimens, sample sizes, and power calculations. To ensure the reliability of these simulations, DTs are rigorously validated against real-world clinical trial data. Furthermore, techniques such as SHapley Additive exPlanations (SHAP) are employed to enhance model transparency and interpretability^24^.

## Enhancing clinical trial efficacy with digital twins

In clinical research, DT technology can enhance trial design by generating precise forecasts of individual patient responses to interventions, enabling the development of more efficient and focused clinical studies.

### Efficacy

Digital twins can improve the efficiency and effectiveness of RCTs^25–27^. They feed the accuracy of individual responses, enforcing targeted treatment decisions through theoretical modeling^28–30^. On the other hand, digital representations can be forecast based on previous interventions and their efficacy data^31^. In silico clinical trials (ISCT) employing digital twins can optimize pharmacological treatments and reduce traditional clinical trial costs, particularly in assisted reproduction^32^. The benefits of integrating digital twins, mobile health, wearable technologies, and AI-driven analytics are shown to improve participant engagement, data collection, and trial efficiency^33^, highlighting a paradigm shift toward more adaptive and patient-centered clinical trials. Moreover, decentralized clinical trials (DCTs) leveraging digital twins can overcome traditional challenges like participant enrollment and retention by integrating remote data collection devices and communication tools^34^.

### Safety assessment

Digital twins significantly enhance drug safety assessments by leveraging comprehensive patient data to predict potential adverse events and individual patient treatment responses^35^. These virtual models can integrate genetic, physiological, and environmental factors to simulate how a patient might react to a specific drug or therapy. This allows researchers to identify possible adverse events before they occur in actual patients, enabling preemptive adjustments to treatment protocols to minimize risks. Additionally, digital twins can facilitate continuous monitoring and real-time analysis, providing deeper insights into the safety profile of interventions across diverse patient populations. By modeling various scenarios and outcomes, digital twins help optimize dosages and reduce the likelihood of adverse events, thereby improving overall patient safety^20,36,37^.

### Drug development

DTs can offer transformative potential in the drug development process by creating highly detailed and dynamic virtual models of patients. These models enable researchers to simulate and predict how new drugs interact with different biological systems, streamlining the drug development pipeline^38^.

- Early-stage discovery: DTs can model disease mechanisms and identify potential therapeutic targets. By simulating the biological processes involved in a disease, researchers gain deeper insights, helping design more effective drugs from the outset.
- Preclinical testing: DTs can mitigate ethical concerns, provide more relevant data predictive of human outcomes, and accurately simulate human responses, making them a viable alternative to or addition to specific animal research.
- Clinical trial simulation: DTs can make virtual cohorts that resemble real-world populations to model clinical trials. These virtual trials try out various doses, treatment plans, and factors for choosing patients. This improves the design of the trials and their chances of success.
- Regulatory submissions: DTs provide loads of information regarding a drug’s safety and effectiveness, which helps simplify regulatory applications. Regulatory agencies like Institutional Review Boards (IRB) play a crucial role in upholding the ethical standards of DT-based trials^39,40^. They consider ethical issues unique to DTs like model bias and transparency in algorithmic decision-making^41^.
- Post-market surveillance: DTs can be continuously updated with data from the real world, allowing them to monitor a medicine’s efficacy and safety after it has been licensed. This guarantees that pharmaceuticals remain safe and effective throughout their lives.

### Sample size and generalization

Large sample sizes are frequently needed in traditional clinical studies to attain statistical power, which can be costly and time-consuming. A possible remedy is provided by digital twins, which allow for more precise treatment result predictions using smaller sample sizes. By simulating virtual patients that accurately reflect real-world diversity and variability, DTs can help identify the minimum number of participants needed to achieve reliable results. This approach reduces the burden on recruitment, shortens trial durations, and lowers costs. Additionally, DTs can perform in silico trials to test various scenarios and refine study designs before actual implementation, ensuring that the physical trial is as efficient and effective as possible^38,42^. Clinical trials have historically struggled to include diverse populations, limiting the understanding of how treatments affect different demographics. Digital twins offer a complementary approach by leveraging existing clinical and real-world data to model patient responses in underrepresented groups. While DTs cannot fully replace direct testing of new treatments, they can help refine trial designs, identify potential variations in treatment effects across populations, and improve the generalizability of clinical research.

### Personalized medicine

The ability of digital twins (DTs) to personalize treatments and enhance precision makes them a valuable tool in medicine^43,44^. By leveraging predictive modeling, DTs can adapt therapies to individual patients’ needs^45,46^. For example, multiple studies have used DTs to predict therapeutic outcomes in cacner^21,22^. By adjusting intervention variables, researchers can simulate patient conditions and optimize treatments while minimizing side effects^27,47^. Also, a personalized heart digital twin was employed to detect substrate abnormalities in scar-dependent ventricular tachycardia and showed outstanding performance; electrogram abnormalities were significantly more frequent in digital twin-predicted sites compared with non-predicted sites^48^.

Current research explores the potential of electronic health records to predict clinical trial outcomes. This involves identifying relationships across diverse medical datasets, even with limited data. By integrating EHR data, researchers can create personalized DTs that closely mimic real-world patient responses, improving trial accuracy. This is particularly useful when clinical data is scarce, allowing for more precise predictions. For instance, Allen et al. used EHR data to simulate stroke patient trajectories, comparing treatment outcomes to placebo scenarios. Using a variational autoencoder (VAE), they generated sequential patient trajectories from a learned low-dimensional embedding space^49^.

### Cost and time issues

A key motivation for integrating DTs in clinical settings is the low success rate of drug development, with only one in ten compounds entering clinical trials ultimately gaining regulatory approval^50^. Phase 1 trials aim to assess a compound’s safety and efficacy as early as possible based on patient data^51^. However, ~80% of trials face delays due to patient enrollment challenges^52^, making it crucial to reduce the number of participants needed for timely evaluations. By augmenting clinical trials with DTs, drug development can be accelerated while minimizing economic and societal burdens. These digital replicas can mimic drug test setups and treatment plans and, thus, significantly reduce the initial phase trial within clinical trials. Therefore, the requirements for resources and time will be decreased by phase simulations^38^.

DTs generate synthetic patient data that can streamline recruitment processes, particularly for rare diseases where patient enrollment is a major bottleneck. Utilizing digitalized technologies and potentially conducting trials with smaller participant groups through DTs, even medications with lower production volumes could become economically viable. In oncology, DTs can simulate comparator arms in Phase 1 and 2 trials, allowing earlier efficacy assessments. Additionally, by generating large-scale simulated datasets, DTs enhance statistical power, enabling faster and more reliable clinical decision-making.

## Ethical advancements with digital twins in RCTs

Digital twins amplify the ethical level of the RCTs in terms of creating more personalized, transparent, and secure solutions. The development of a virtual clinical trial system aligns with ensuring patient safety is improved due to risk minimization by providing predictive insights^47^, increasing the patients’ well-being^20,53^. Digital replicas significantly benefit researchers in acquiring predictive knowledge. This knowledge enables the proactive recognition and mitigation of potentially hazardous threats rather than a reactive approach of waiting for and addressing risks as they arise. Therefore, patients’ willingness to sign on to research protocols by being given clear instructions about risks and benefits improves their understanding^54^.

Additionally, they help achieve equality and inclusivity by involving an active population during trial design, reducing healthcare disparities^55^. Raw data from digital twins is meticulously obtained, representing transparency and avoiding the lack of confidence among the masses^56^. Along with strengthening personal autonomy and awareness, digital replicates help accelerate equality and allow for more excellent representation in clinical research. Digital twins ensure that trial designs are refined to address the specific requirements and characteristics of various populations and reduce healthcare disparities. Hence, this impressive dedication to equitable clinical trials is respected by its ethical alignment and the establishment of equal opportunity for health. However, integration of these technologies presents several complexities, including multifaceted ethical and privacy considerations. Therefore, strict regulations must govern data management to preserve sensitive information and maintain privacy.

### Informed consent and trust

Constructing a DT-enabled clinical trial requires continuous, real-time data collection and analysis. While informed consent is a fundamental requirement in all clinical research, the complexity and scale of data involved in DT-enabled trials make robust, transparent consent procedures even more critical^33^. These studies often rely on advanced computational models, including deep learning, which may lack full explainability. As a result, participants must be informed not only about what data is collected, but also how it is transmitted, analyzed, and used to simulate clinical outcomes.

Digital twins contribute to patient-centered trials that boost patients’ informed consent. By providing exhaustive information on the possible risks and benefits associated with the study, digital twins assist trial participants in weighing the pros and cons of involvement in the study, enabling them to make informed decisions.

Digital twins can transform informed-consent discussions from abstract risk tables to personalized, visual narratives^56^. At Johns Hopkins, a genotype-specific cardiac-twin program creates a 3D replica of each arrhythmia patient using MRI and genomic data, then runs simulations that show how catheter ablation might change that person’s ventricular-tachycardia circuits; the color-coded playback makes benefits and residual risks tangible for candidates considering enrollment in an ongoing trial^57^. Additionally, evidence that such visual tools improve understanding is supported by a randomized study of an interactive\multimedia consent platform. Participants who used the digital interface reported higher satisfaction, greater ease of use, and faster completion compared to those who used paper forms, while still maintaining high levels of comprehension^58^.

Moreover, Sinisi et al. demonstrated how continuous-time monitors embedded within virtual physiological human (VPH) models enabled modeling of bounded safety of treatments in clinical trials, offering greater transparency in assessing trial risk thresholds^32^. As clinical trials increasingly become decentralized and cross state or national borders, through site-less designs and virtual visits, they introduce new regulatory challenges. These include differences in state-level oversight and telehealth licensing requirements. DT-enabled RCTs, may consist of virtual visits across state lines or national borders rather than at a specific physical study site^59^.

Taken together, these experiences suggest that patient-specific digital twins, paired with interactive consent software, can enhance autonomy and promote genuinely informed, patient-centered participation in trials.

### Privacy and data security

A significant challenge in digital twin technology is ensuring data privacy and security. Digital twins rely on vast amounts of data, including personal health records, genetic information, and lifestyle details, raising critical concerns about unauthorized access, data breaches, and misuse. The highly detailed and dynamic nature of digital twins, which often incorporate behavioral, environmental, and biological information, raises significant re-identification risks even in ostensibly anonymized datasets. Furthermore, the ability of digital twins to predict sensitive future health outcomes introduces ethical and legal complexities, as unauthorized disclosure could impact insurability or employment. Aggregating data from multiple systems also exposes digital twin infrastructures to interoperability vulnerabilities. Additionally, because digital twins evolve over time, maintaining valid informed consent becomes increasingly challenging, requiring ongoing engagement with data subjects to ensure ethical transparency throughout the digital twin lifecycle.

To address these risks, compliance with data-protection regulations such as the GDPR and HIPAA is essential^60^. Real-world deployments demonstrate how this is already being achieved. Twin Health’s whole-body digital twin platform encrypts all protected-health information, operates within a HIPAA-compliant cloud, and requires Business Associate Agreements (BAAs) with every vendor, embedding privacy safeguards throughout its workflow^61^. Similarly, a peer-reviewed digital-twin framework for real-time monitoring reports an end-to-end encrypted architecture that fully aligns with such data-protection regulations, providing a research-grade template for regulatory compliance^62^. Collectively, these examples show that rigorous technical controls (encryption, role-based access, vendor BAAs) and organizational measures (independent audits, risk assessments) can translate legal mandates into everyday digital-twin practice.

Robust security measures, including advanced encryption, secure storage solutions, and strict access controls, play a vital role in safeguarding sensitive information. Additionally, de-identification techniques are crucial in anonymizing data while preserving its usability for researchers. Equally important is the rigorous testing of the applications and interfaces used in the digital twin. This testing, not only from the software perspective but also from the security perspective^63^, ensures that they are bug-free and less vulnerable, instilling confidence in the reliability of digital twin technology. In decentralized clinical trials, data management from privacy and security is crucial, as such data is collected and processed across different platforms and devices with varying levels of trust and security.

Recent advancements in generative AI offer promising solutions for enhancing privacy in digital twins used in clinical trials. Models like TWIN-GPT, for example, employ three key privacy risk evaluation methods: presence disclosure, attribute disclosure, and nearest neighbor adversarial, to detect data leakage and assess vulnerabilities in the event of a cyberattack^42^. While a detailed discussion of these methods is beyond the scope of this review, it is important to note that these metrics evaluate the likelihood of an attacker identifying which features belong to specific patients. By utilizing synthetic data generated by the LLM model in this study, the risk of such privacy breaches is significantly reduced.

### Reducing animal use

DT testing is most valuable before traditional human efficacy trials begin, that is, during the preclinical and Phase 0/first-in-human micro-dosing stages, where most exploratory animal studies are currently conducted. In these early phases, investigators need pharmacokinetic, toxicity, and dose-finding data rather than conclusive proof of clinical benefit; DTs can provide that information by simulating organ- and system-level responses to candidate compounds. If the virtual models predict an acceptable safety margin, researchers can proceed directly to a small Phase I study in healthy volunteers, thus replacing or significantly reducing animal use. Later-phase trials (Phase II–IV) already involve human participants, hence DTs at those stages serve different roles, such as creating synthetic control arms or enabling patient-specific dose optimization, while the animal-replacement benefit is mainly realized earlier in preclinical decision-making. In this way, digital-twin methodology supports the 3Rs principle (replacement, reduction, refinement), where it is most important for animal welfare, without disrupting the regulatory sequence of human trials^38,64,65^.

### Equity and inclusivity

DTs can promote more significant equity and inclusivity in clinical trials by ensuring that diverse populations are adequately represented. Traditional trials often struggle with recruiting a representative sample of participants, leading to disparities in the data and potential biases in the results. While DTs cannot replace real-world testing, they can simulate diverse patient populations, including those from underrepresented groups, ensuring that the effects of treatments are studied across a wide range of individuals. This inclusivity helps develop effective therapies for all population segments, addressing health disparities and promoting equity in healthcare.

### Real-time monitoring and ethical oversight

DTs allow for continuous real-time monitoring of patient responses during clinical trials. This capability enables researchers to promptly detect and respond to adverse events, enhancing patient safety. Additionally, real-time monitoring supports ethical oversight by providing ongoing data to ethics committees and regulatory bodies, ensuring that the trial adheres to ethical standards throughout its duration. This proactive approach to monitoring and oversight strengthens the ethical framework of clinical trials, ensuring that participant welfare remains a priority. For instance, a study utilized a transfer-learned digital twin model to develop a nurse-in-the-loop predictive control system for managing glucose levels and weight in patients with type 2 diabetes^66^. This intelligent system continuously gathers daily updates on patients’ diets, ensuring strict monitoring and minimizing adverse effects during the clinical trial.

### Transparency and accountability

Integrating DTs in clinical trials promotes transparency and accountability. By providing detailed simulations and predictive models, DTs allow researchers to justify their decisions and treatment plans with robust data. This transparency fosters a culture of accountability, as researchers can be held to higher standards of evidence and rationale for their actions. Furthermore, detailed documentation of DT simulations and outcomes can be shared with participants, ethics committees, and regulatory bodies, enhancing the overall transparency of the research process.

### Digital twins versus alternative approaches

While digital twins offer significant promise through real-time, individualized modeling, it is essential to place them within the broader landscape of existing methods. Synthetic control methods, widely used in causal inference studies, construct a weighted combination of untreated units to approximate the counterfactual for a treated entity^67^. These methods are computationally less intensive and provide a robust approach for retrospective analysis. However, they lack the dynamic adaptability and continuous personalization that DTs offer, especially in complex and evolving systems where real-time feedback and adjustment are critical.

Similarly, simulation-based approaches such as agent-based models can capture the interactions of multiple agents within a system and predict aggregate behavior^68^. While they are powerful for studying population-level dynamics, these models generally generalize across groups instead of tailoring predictions to individual entities. In contrast, DTs uniquely integrate live data streams to maintain a high-fidelity, evolving representation of a specific object, patient, or process. This distinction highlights the added value of DTs in contexts that require personalized, dynamic, and predictive modeling beyond what traditional synthetic or simulation-based techniques can achieve.

## Regulatory and implementation challenges

There are two continuums of the positive impacts of digital twins in RCTs that come with the integration process: the problems of regulations and implementation. Two issues are how the practical measures and technological needs for digital twins in RCTs are solved, and how the implementation process will be undertaken. The process involves not just the choice of fitting technology platforms but also designing concerted data collection, analysis, and interpretation methods^69^. The incorporation of digital twins in clinical trials necessitates a robust technological infrastructure. High-fidelity computational models must accurately replicate human physiology and disease states. These models require extensive data inputs from various sources, including medical imaging, genomics, real-time health monitoring devices or any medical-purpose Internet of Things (IoT) devices, facilitating quick analysis and response to conditions.

High-performance computing resources and advanced algorithms, particularly those involving artificial intelligence and machine learning, are crucial for processing and analyzing these large datasets. Integrating digital twins into clinical trials requires seamless interoperability between diverse health information systems. This includes exchanging data across electronic health records, mobile health applications, and wearable devices. Standardization of data formats and communication protocols, such as Health Level Seven (HL7) and Fast Healthcare Interoperability Resources (FHIR), ensures that different systems can work together effectively. Moreover, cloud computing platforms provide scalable solutions to keep and handle the extensive amounts of data generated by digital twins, facilitating real-time data access and collaboration among researchers worldwide^70^. In addition, synergistic work within the regulative bodies, the researchers, and the relevant industry professionals is crucial for adequately smoothening the regulatory procedures and the smooth introduction of digital twins into the clinical trial processes.

### Regulatory frameworks and compliance

Implementing well-defined regulatory structures fosters accountability and promotes compliance with established protocols^23^. Critical regulatory regime gaps must be widely recognized and appropriately settled to accord the ethical use of digital twins in clinical trials^38^. For example, DTs often fall under the category of software as a medical device (SaMD), yet existing regulatory pathways may not fully capture their dynamic and adaptive nature^71^. Therefore, regulators need to develop specific frameworks that address the unique characteristics of DTs. Digital twins must be practically incorporated into RCTs within a specific and standard framework to overcome every challenge^72^. Adherence to established frameworks governing healthcare, data privacy, medical devices, and artificial intelligence is obligatory. One of the most critical factors is ensuring that digital twins are used ethically and responsibly during clinical trials; consequently, covering regulatory gaps is imperative^71^. In addition, the regulatory landscape surrounding digital twin development is multifaceted, reflecting the novelty of the technology and the consequent lack of well-defined regulatory guidance. Stakeholders may develop regulations covering all digital twin applications in RCTs, revealing that some existing rules may not suit digital twin usage. Such development can trigger recommendations for adjustments or the inclusion of new guidelines.

### Bias assessment

There are concerns regarding applying AI algorithms. Biases stemming from gender, ethnicity, culture, politics, ideology, and language within the training data significantly contribute to potential biases in clinical and research settings^73^. The potential for bias exists within these models due to inherent limitations in the training datasets. A proactive approach by diversifying data sources and implementing continuous model updates mitigates these biases. In addition, DTs do not inherently eliminate bias; rather, their accuracy and fairness depend significantly on the quality, diversity, and representativeness of the data used to construct them. Key considerations include evaluating sampling methods, identifying underrepresented groups, and assessing systematic missingness or measurement bias. In cases where fully representative data are unavailable, techniques such as reweighting, oversampling minority groups, or incorporating synthetic data generation may help mitigate bias^74^. Nevertheless, residual biases should be explicitly acknowledged to maintain transparency and ethical integrity in digital twin development. Assigning liability for errors arising from digital predictions or diagnoses presents a significant challenge. Additionally, DTs frequently rely on AI and machine learning algorithms, which can function as opaque “black boxes.” There is a lack of standardized methods for validating these models’ clinical performance, interpretability, and reliability^75^. Without rigorous validation protocols, there is a risk of deploying biased or unproven models in clinical settings. Establishing well-defined legal frameworks is crucial to delineating the responsibilities of healthcare providers, technology developers, and all relevant stakeholders in the event of inaccurate or erroneous outcomes^53^.

### Equity and accessibility

Ensuring equitable access to DT technology is vital to avoid exacerbating healthcare disparities^76^. Barriers such as access to necessary technology and infrastructure, especially in low-resource settings, need to be addressed. Training and educational programs should be available to healthcare providers and researchers to equip them with the skills to use DT technology. Financial support and incentives may be necessary to ensure all healthcare organizations can adopt and benefit from digital twin technology^77^.

## Future directions

The future of DTs in clinical trials is promising, with several key areas poised for development and enhancement. One primary focus is conducting pilot studies to validate the efficacy and reliability of DTs across various therapeutic areas and patient populations. These studies will compare outcomes from traditional trials and those utilizing DTs. They will provide robust evidence to support their broader adoption and demonstrate their potential to enhance trial design, patient safety, and overall efficacy.

Interdisciplinary collaboration is crucial for the successful implementation of DTs. Researchers, clinicians, data scientists, and regulatory bodies must work together to address the technical, ethical, and regulatory challenges associated with DTs. Establishing interdisciplinary research teams and consortia will facilitate the sharing of knowledge, resources, and best practices, accelerating the development of standardized protocols and improving system interoperability. In addition, ensuring the ethical deployment of DTs involves engaging patients in the development and implementation process. Transparency about how DTs will be used, potential risks and benefits, and data-protection measures are essential to build trust. Involving patient advocacy groups and ethics committees can help address ethical concerns and promote patient-centered approaches. Feedback from patients can guide the refinement of DT models to meet their needs and expectations better. Also, education and training programs for healthcare professionals, researchers, and regulatory personnel are vital as DTs become more prevalent in clinical research. These programs should cover the principles and applications of digital twin technology, data management, ethical considerations, and regulatory compliance. Educational initiatives should raise awareness about the benefits and potential of DTs among the broader public and patient communities.

Advancements in AI and machine learning will play a critical role in enhancing the capabilities of DTs, enabling more accurate simulations, better predictive modeling, and more efficient data processing. These advancements will also help address challenges related to bias and fairness, ensuring that DT models are robust and equitable.

Regulatory frameworks must evolve to keep pace with digital twin technology advancements. Regulatory bodies should work closely with researchers and industry stakeholders to develop guidelines that ensure DTs’ safe and ethical use in clinical trials. This includes establishing standards for model validation and verification, data privacy and security, and addressing ethical considerations related to informed consent and patient rights. Proactive regulatory evolution will facilitate the responsible integration of DTs into clinical research. Developing global standards and ensuring interoperability across healthcare systems and technologies are essential for fully realizing the potential of DTs. International collaboration is needed to establish common data formats, modeling standards, and validation criteria, enabling seamless integration of DTs worldwide. Harmonizing these standards will facilitate data sharing, enhance scalability, and promote the global adoption of DT technology.

Digital-twin platforms constantly transmit imaging, sensor, and genomic data to trial databases, making them attractive targets for cyber attackers^78,79^. In 2020, a ransomware attack on eResearch Technology locked investigators out of e-clinical systems, forcing several COVID-19 trials back to paper logs, which delayed enrollment and analysis^80^. To defend against such threats, some sponsors now conduct offensive security drills: digital twins of their networks undergo simulated firewall, routing-change, and breach scenarios so vulnerabilities can be addressed before going live^80^. Complementary defensive measures, including real-time anomaly detection and access monitoring, then safeguard live patient data during the trial.

Blockchain can serve as an experimental countermeasure^81,82^. A blockchain is an append-only, distributed ledger where each record is cryptographically linked to the previous one and validated by network consensus^83^. A breast cancer pilot demonstrated that logging every eCRF transaction on a private Ethereum chain prevented data tampering and remained operational during a regional cloud outage with zero downtime^84^. Permissioned (private) chains also eliminate a single point of failure and provide regulators with an immutable audit trail, but they raise practical and ethical questions. One issue is about clinician usability as blockchain operates beneath familiar electronic data capture (EDC) front-ends, but site staff still require training and governance support. Another matter is the right to withdraw. This is because immutability conflicts with participants’ right to delete their data; hybrid models that store PHI off-chain behind revocable encryption keys are being trialed. Finally, scalability and throughput constraints must be benchmarked against high-volume DT telemetry before large pivotal trials.

Moving forward, comparative assessments should determine whether these architectures meet HIPAA/GDPR requirements and protect participants’ withdrawal rights, key obstacles before blockchain can transition from a proof-of-concept to routine use.

## Conclusion

Digital twins have the potential to significantly enhance clinical research by optimizing trial designs, improving patient safety, and advancing personalized medicine through predictive modeling. While they offer promising avenues for reducing trial costs and durations, their successful implementation requires rigorous validation to address challenges such as technological infrastructure, regulatory frameworks, data reliability, security, privacy, and equitable access. Importantly, DTs should not be seen as replacements for traditional concurrent controls but rather as complementary tools to refine trial design and improve representation in clinical research. While they promise reduced costs and durations for clinical trials, their successful integration requires addressing technological infrastructure, regulatory frameworks, data quality, data security and privacy, and equitable access challenges. Interdisciplinary collaboration and AI and machine learning advancements are crucial for their development. Having anticipated and acted ahead through the regulatory gaps and cleverly looking at digital twins’ technology, stakeholders can effectively determine how to exploit its transformative characteristics. This will lead to a revolution in using RCTs to improve patient prognosis. Coordinated activities of regulatory agencies, researchers, and industries will have to be in place to develop digital twins to overcome these challenges. Digital twins can be a constructive part of these ethical principles and patient safety if attention is given; hence, it empowers future innovative and advanced research clinical trial methodologies and assist in a more efficient and effective clinical trial.

## Data Availability

No datasets were generated or analysed during the current study.
