# Author Correction: Enhancing randomized clinical trials with digital twins

**DOI:** 10.1038/s41540-025-00609-8

**Published:** 2025-11-06

**Authors:** Hossein Akbarialiabad, Amirmohammad Pasdar, Dédée F. Murrell, Mehrnaz Mostafavi, Farhan Shakil, Ehsan Safaee, Sancy A. Leachman, Alireza Haghighi, Michelle Tarbox, Christopher G. Bunick, Ayman Grada

**Affiliations:** 1 University of Utah, Salt Lake City, UT USA; 2 Department of Dermatology, St George Hospital, Sydney, NSW Australia; 3 Faculty of Medicine, UNSW Medicine, University of New South Wales, Sydney, NSW Australia; 4 Missouri Baptist Medical Center, St. Louis, MO USA; 5 Washington University in St. Louis School of Medicine, St. Louis, MO USA; 6 School of Computing and Information Systems, University of Melbourne, Melbourne, VIC Australia; 7 Faculty of Allied Medicine, Shahid Beheshti University of Medical Sciences, Tehran, Iran; 8 George Herbert Walker School of Business & Technology, Webster University, Saint Louis, MO USA; 9 Faculty of Medicine, Shahed University, Tehran, Iran; 10 Division of Genetics, Department of Medicine, Brigham and Women’s Hospital, Harvard Medical School, Boston, MA USA; 11 Department of Medicine, Brigham and Women’s Hospital, Harvard Medical School, Boston, MA USA; 12 The Broad Institute, Cambridge, MA USA; 13 Department of Dermatology, Texas Tech University Health Sciences Center, Lubbock, TX USA; 14 Department of Dermatology and Program in Translational Biomedicine, Yale School of Medicine, New Haven, CT USA; 15 Department of Dermatology, Case Western Reserve University School of Medicine, Cleveland, OH USA

**Keywords:** Diseases, Business and industry

Correction to: *npj Systems Biology and Applications*; 10.1038/s41540-025-00592-0, published online 03 October 2025

In this article the author name Christopher G. Bunick was incorrectly written as Chritopher G. Bunick. The original article has been corrected.

